# Comparison of chemoradiotherapy and gemcitabine plus nab-paclitaxel for locally advanced pancreatic cancer: an integrated analysis of two randomized phase II trials (JCOG2408A)

**DOI:** 10.1186/s12885-026-15699-8

**Published:** 2026-02-10

**Authors:** Yusuke Sano, Riku Kajikawa, Junki Mizusawa, Tatsuya Ioka, Masato Ozaka, Satoaki Nakamura, Yoshinori Ito, Junji Furuse, Satoshi Kobayashi, Haruhiko Fukuda, Takuji Okusaka, Masafumi Ikeda, Haruo Miwa, Naoki Sasahira, Fumio Nagashima, Kazuyoshi Ohkawa, Kentaro Yamazaki, Masashi Kanai, Taro Yamashita, Kazuo Hara, Yukiko Takayama, Yoshito Komatsu, Nao Fujimori, Naoki Hama, Ken Kamata, Terumasa Hisano, Satoshi Shimizu, Kazutoshi Tobimatsu, Shin Maeda, Makoto Ueno

**Affiliations:** 1 Japan Clinical Oncology Group Data Center/Operations Office, National Cancer Center Hospital, Tokyo, Japan; 2 Department of Gastroenterology, Yokohama City University Graduate School of Medicine, Yokohama, Japan; 3 Department of Oncology Center, Yamaguchi University Hospital, Yamaguchi, Japan; 4 Department of Hepato-Biliary-Pancreatic Medicine, Cancer Institute Hospital of Japanese Foundation for Cancer Research, Tokyo, Japan; 5 Division of Radiation Oncology, Kansai Medical University Hospital, Osaka, Japan; 6 Department of Radiation Oncology, Showa Medical University School of Medicine, Tokyo, Japan; 7 Department of Gastroenterology, Kanagawa Cancer Center, Yokohama, Japan; 8 Department of Hepatobiliary and Pancreatic Oncology, National Cancer Center Hospital, Tokyo, Japan; 9 Department of Hepatobiliary and Pancreatic Oncology, National Cancer Center Hospital East, Kashiwa, Japan; 10 Gastroenterological Center, Yokohama City University Medical Center, Yokohama, Japan; 11 Department of Medical Oncology, Kyorin University Faculty of Medicine, Tokyo, Japan; 12 Department of Hepatobiliary and Pancreatic Oncology, Osaka International Cancer Institute, Osaka, Japan; 13 Division of Gastrointestinal Oncology, Shizuoka Cancer Center, Shizuoka, Japan; 14 Department of Clinical Oncology, Kansai Medical University Hospital, Osaka, Japan; 15 Department of Gastroenterology, Kanazawa University School of Medicine, Kanazawa, Japan; 16 Department of Gastroenterology, Aichi Cancer Center Hospital, Nagoya, Japan; 17 Department of Internal Medicine, Institute of Gastroenterology, Tokyo Women’s Medical University, Tokyo, Japan; 18 Department of Cancer Center, Hokkaido University Hospital, Sapporo, Japan; 19 Department of Medicine and Bioregulatory Science, Graduate School of Medical Sciences, Kyushu University, Fukuoka, Japan; 20 Department of Hepatobiliary and Pancreatic Surgery, National Hospital Organization Osaka National Hospital, Osaka, Japan; 21 Department of Gastroenterology and Hepatology, Faculty of Medicine, Kindai University, Osaka, Japan; 22 Department of Hepato-Biliary-Pancreatology, National Hospital Organization Kyushu Cancer Center, Fukuoka, Japan; 23 Department of Gastroenterology, Saitama Cancer Center, Saitama, Japan; 24 Division of Gastroenterology, Department of Internal Medicine, Kobe University Graduate School of Medicine, Kobe, Japan; 25 Japan Clinical Oncology Group Data Center/Operations Office, National Cancer Center Hospital, 5-1-1 Tsukiji, Chuo-ku, Tokyo, 104-0045 Japan

**Keywords:** Locally advanced pancreatic cancer, Chemoradiotherapy, Systemic chemotherapy, Gemcitabine plus nab-paclitaxel

## Abstract

**Background:**

Two main therapeutic approaches are currently used for locally advanced pancreatic cancer (LAPC): chemoradiotherapy and systemic chemotherapy. It remains unclear which approach may be more promising, or whether these strategies should be considered alternative or complementary therapeutic options in the management of LAPC. Clinical outcomes and safety were assessed for S-1 plus concurrent radiotherapy (S-1 + RT) and gemcitabine plus nab-paclitaxel (GnP) in patients with LAPC.

**Methods:**

We conducted a pooled exploratory analysis of individual patient data derived from two multi-institutional randomized phase II trials conducted by the Japan Clinical Oncology Group (JCOG1106 and JCOG1407). JCOG1106 evaluated S-1 + RT with or without induction chemotherapy. JCOG1407 compared GnP with modified FOLFIRINOX. Based on the results of these trials, S-1 + RT and GnP were selected as promising regimens for chemoradiotherapy and systemic chemotherapy, respectively. The primary endpoint of this study was progression-free survival (PFS). Inverse probability of treatment weighting (IPTW) with stabilized weights was applied based on the propensity score to account for baseline imbalances between the two groups.

**Results:**

A total of 113 patients were included. After adjustment for patient characteristics, Kaplan–Meier curves showed median PFS, overall survival (OS), and distant metastasis-free survival (DMFS) of 10.2 vs. 9.3 (hazard ratio [HR], 0.88; 95% confidence interval [CI], 0.60–1.30), 19.1 vs. 21.2 (HR, 0.73; 95% CI, 0.48–1.11), and 11.5 vs. 13.1 months (HR, 0.73; 95% CI, 0.49–1.08) for S-1 + RT and GnP, respectively. Treatment received after protocol therapy differed substantially: 77.5% in the S-1 + RT group received single-agent chemotherapy, whereas 50.0% in the GnP group, received more intensive regimens, including multi-agent chemotherapy or chemoradiotherapy.

**Conclusions:**

GnP may offer advantages in suppressing micrometastatic disease, whereas S-1 + RT may provide benefits in local disease control. These findings suggest that both approaches represent important and complementary therapeutic options for LAPC. Prospective randomized studies are warranted to determine the optimal initial strategy.

**Supplementary Information:**

The online version contains supplementary material available at 10.1186/s12885-026-15699-8.

## Introduction

Pancreatic cancer is the sixth leading cause of cancer-related deaths worldwide, with approximately 510,000 new cases and 470,000 deaths reported globally in 2022 [1]. Approximately 80–85% of pancreatic cancers are unresectable, with the disease being locally advanced or metastatic at diagnosis. At least one-third of the patients with pancreatic cancer present with locally advanced disease due to extensive vascular involvement, which precludes surgical resection [2]. Patients with locally advanced pancreatic cancer (LAPC) are treated with chemoradiotherapy or systemic chemotherapy. However, the optimal initial approach for patients with LAPC remains controversial.

The Hepatobiliary and Pancreatic Oncology Group of Japan Clinical Oncology Group (JCOG) conducted two selection design randomized phase II trials in patients with LAPC to identify the most promising chemoradiotherapy and systemic chemotherapy regimens. JCOG1106 was conducted to compare the efficacy and safety of S-1 and concurrent radiotherapy (S-1 + RT), with and without the induction of gemcitabine, to determine the most promising chemoradiotherapy regimen. JCOG1106 reported that S-1 + RT without induction gemcitabine had more promising efficacy with longer OS (hazard ratio [HR] for S-1 + RT with induction gemcitabine, 1.26; 95% confidence interval [CI], 0.82–1.93) [3]. JCOG1407 was conducted to compare the efficacy and safety of gemcitabine plus nab-paclitaxel (GnP) with modified 5-fluorouracil, leucovorin, irinotecan, and oxaliplatin (FOLFIRINOX). In the JCOG1407 trial, the 1-year OS of the primary endpoint was marginally better with GnP than with modified FOLFIRINOX, whereas this trend was reversed in the subsequent period (1-year OS, 82.8% vs. 77.4%; 2-year OS, 40.6% vs. 46.2%). GnP showed better efficacy in terms of response rate (42.1% vs. 30.9%), disease control rate (96.5% vs. 87.3%), carbohydrate antigen 19–9 (CA19–9) response rate (85.0% vs. 57.1%), and reduced gastrointestinal toxicity. Consequently, GnP was selected as the more promising regimen for the subsequent phase III trial [4].

Findings from these two independent phase II trials suggest that S-1 + RT without induction of gemcitabine and GnP represent reasonable candidates for further development as standard approaches for LAPC. However, no randomized phase III trials have directly compared these two approaches. Several retrospective cohort studies have been reported; however, these are limited by a lack of standardized treatment regimens and assessment procedures [5–7]. As a result, it remains unclear which approach is more promising, or whether these strategies should be considered alternative or complementary therapeutic options in the management of LAPC. A randomized comparison of S-1 + RT and GnP would be informative; however, such a trial is challenging owing to the differences in treatment modalities and the duration required to evaluate the outcomes. In this study, clinical outcomes and safety were assessed for patients with LAPC treated with S-1 + RT or GnP using data from JCOG1106 and JCOG1407.

## Methods

### Study design and patients

This exploratory analysis was a pooled analysis using data from JCOG1106 and JCOG1407. We had full access to the trial data for JCOG1106 and JCOG1407. Each trial was a randomized phase II trial for a subsequent phase III trial comparing chemoradiotherapy with systemic chemotherapy in patients with LAPC. The design and conduct of JCOG1106 and JCOG1407 have been reported in detail elsewhere [3, 4]. Briefly, JCOG1106 evaluated S-1–based chemoradiotherapy strategies, whereas JCOG1407 compared GnP with modified FOLFIRINOX as systemic chemotherapy. For the present analysis, patients assigned to S-1 + RT in JCOG1106 and those assigned to GnP in JCOG1407 were included.

The protocol for this exploratory study (JCOG2408A) was approved by the JCOG Protocol Review Committee and the Institutional Review Board of National Cancer Center, Tokyo.

### Endpoints

The primary endpoint of this exploratory analysis was progression-free survival (PFS), which was defined as the time from the date of registration to the date of disease progression or death from any cause. Data were censored on the last day when the patient was alive without any evidence of progression. JCOG1106 was conducted earlier than JCOG1407, and the shorter OS observed in the S-1 + RT group may have been influenced by the limited availability of post-progression treatments at the time. Therefore, PFS was selected as the primary endpoint in this study to enable a more accurate comparison between S-1 + RT and GnP.

Secondary endpoints were OS, distant metastasis-free survival (DMFS), and incidence of adverse events. OS was defined as the time from the date of registration to the date of death from any cause, and data were censored on the last day the patient was alive. DMFS was defined as the time from the date of registration to the date of distant metastasis or death from any cause. Data were censored on the last day when the patient was alive without any evidence of distant metastasis. Toxicity was evaluated according to the Common Terminology Criteria for Adverse Events version 4.0.

### Statistical analysis

Differences in the distribution of baseline characteristics between the S-1 + RT and GnP groups were assessed using the standardized mean difference (SMD), with an absolute SMD < 0.1, indicating an acceptable balance between the two groups [8]. The Kaplan–Meier method was used to estimate crude PFS, OS, and DMFS. Hazard ratios (HR) and 95% confidence intervals (CI) with robust variance were estimated using the Cox proportional hazards model. Inverse probability of treatment weighting (IPTW) with stabilized weights was applied based on the propensity score to account for baseline imbalances between the two groups, as this approach minimizes patient loss compared with matching methods and reduces the impact of extreme weights, allowing inclusion of patients with propensity scores close to 0 or 1 [9–12]. IPTW was used to estimate the average treatment effect in the overall study population. The propensity score for each patient was calculated as the probability of receiving GnP, using a logistic regression model that included the following covariates: age (< 65 vs. ≥65), sex (male vs. female), Eastern Cooperative Oncology Group performance status (ECOG PS) (0 vs. 1), primary tumor location (pancreatic head vs. pancreatic body or tail), lymph node metastasis (N0 vs. N1), invasion of celiac artery (no vs. yes), invasion of superior mesenteric artery (no vs. yes), CA19–9 (< 1,000 U/mL vs. ≥1,000 U/mL), serum albumin (< 4.0 mg/dL vs. ≥4.0 mg/dL), and C-reactive protein (CRP) (< 0.3 mg/dL vs. ≥0.3 mg/dL). Stabilized weights were calculated as *P(T = 1)/PS* for patients in the GnP group and *(1 − P(T = 1))/(1 − PS)* for patients in the S-1 + RT group, where *T* denotes the group indicator (*T = 0*: S-1 + RT, *T = 1*: GnP) and *PS* denotes the propensity score. The balance of covariates between the two groups after adjustment was also assessed using the SMD. PFS, OS, and DMFS were estimated using the adjusted Kaplan–Meier method. Adjusted HR and 95% CI with robust variance were estimated using the Cox proportional hazards model.

Several sensitivity analyses, including the IPTW method (without stabilization), were conducted to evaluate the robustness of the estimated treatment effects. Patients with extreme propensity scores (< 0.1 or > 0.9) were excluded. Additionally, propensity score matching was performed using 1:1 greedy matching without replacement, with a caliper of width equal to 0.2 of the pooled standard deviation of the logit of the propensity score [13]. Stratified Cox regression by propensity score quintile was used to estimate the HR and 95% CIs [14]. Regression adjustment was also performed by including the propensity score as a covariate in the model. In addition, multivariable analysis was performed using a Cox regression model, with the treatment group and baseline characteristics (listed in Table 1) included as covariates, to estimate the HR and 95% CIs. All statistical analyses were performed using SAS software, version 9.4 (SAS Institute Inc. Cary, NC, USA).

## Results

### Patient characteristics

**Fig. 1:**
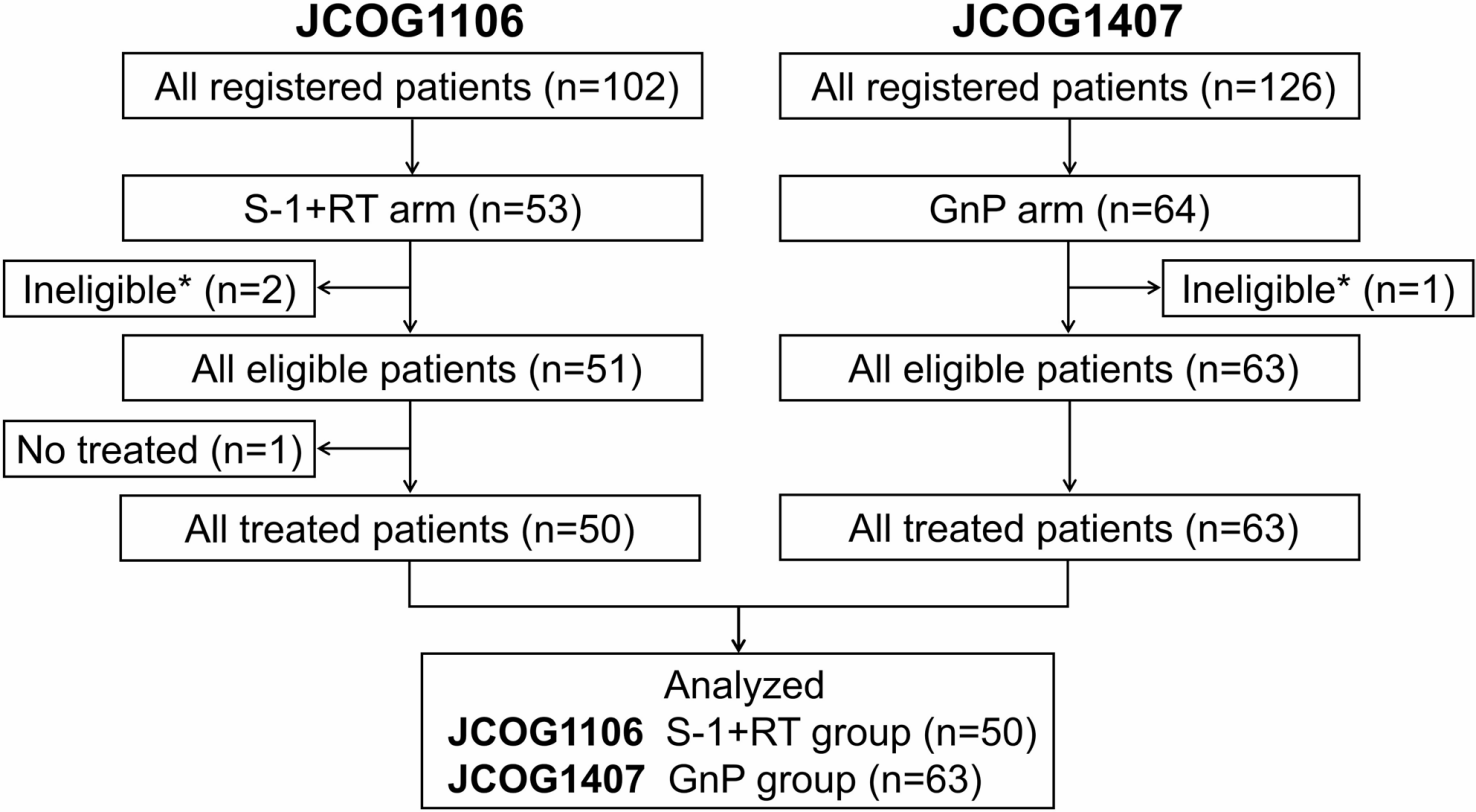
Patient flow diagram Ineligible*: not meeting the inclusion criteria of JCOG1106 or JCOG1407 S-1 + RT, S-1 with concurrent radiotherapy; GnP, gemcitabine plus nab-paclitaxel

Of the 53 patients who were assigned to receive S-1 + RT in JCOG1106, three patients were excluded. Two patients were ineligible due to distant metastasis before the start of the protocol treatment, and one patient could not commence treatment due to liver infection. One of the 64 patients assigned to receive GnP in JCOG1407 was excluded because the baseline serum creatinine level was missing. Fifty patients from JCOG1106 and 63 patients from JCOG1407 were included in the present analysis (Fig. 1).

The unadjusted patient characteristics of the S-1 + RT and GnP groups are presented in Table 1. Several baseline variables differed between the two treatment groups, indicating potential imbalance prior to propensity score adjustment. The proportion of females was higher in the S-1 + RT group than in the GnP group (56.0% vs. 39.7%). There was a trend toward more patients in the S-1 + RT group with findings suggestive of more advanced disease or poorer physical condition, such as higher frequencies of lymph node metastasis (22.0% vs. 15.9%), invasion of superior mesenteric artery (82.0% vs. 65.1%), lower serum albumin (< 4.0 mg/dL, 38.0% vs. 31.8%), and higher CRP (≥ 0.3 mg/dL, 38.0% vs. 27.0%).

**Table 1:** Unadjusted patient characteristics

	S-1 + RT (n=50)		GnP (n=63)		SMD
Age, years					
Median (range)	65	(38–78)	66	(47–75)	0.134
< 65	22	(44.0)	27	(42.9)	0.023
≥ 65	28	(56.0)	36	(57.1)	
Sex					0.331
Male	22	(44.0)	38	(60.3)	
Female	28	(56.0)	25	(39.7)	
ECOG PS					0.031
0	35	(70.0)	45	(71.4)	
1	15	(30.0)	18	(28.6)	
Primary tumor location					0.081
Pancreatic head	29	(58.0)	34	(54.0)	
Pancreatic body or tail	21	(42.0)	29	(46.0)	
Lymph node metastasis					0.157
N0	39	(78.0)	53	(84.1)	
N1	11	(22.0)	10	(15.9)	
Invasion of celiac artery					0.058
No	20	(40.0)	27	(42.9)	
Yes	30	(60.0)	36	(57.1)	
Invasion of superior mesenteric artery					0.391
No	9	(18.0)	22	(34.9)	
Yes	41	(82.0)	41	(65.1)	
CA19–9, U/mL					0.152
< 1,000	39	(78.0)	45	(71.4)	
≥ 1,000	11	(22.0)	18	(28.6)	
Serum albumin, mg/dL					0.132
< 4.0	19	(38.0)	20	(31.7)	
≥ 4.0	31	(62.0)	43	(68.3)	
CRP, mg/dL					0.237
< 0.3	31	(62.0)	46	(73.0)	
≥ 0.3	19	(38.0)	17	(27.0)	

Data are number (%) unless stated otherwise

*S-1 + RT* S-1 with concurrent radiotherapy, *GnP* Gemcitabine plus nab-paclitaxel, *SMD* Standardized mean difference, *ECOG PS* Eastern Cooperative Oncology Group performance status, *CA19–9* Carbohydrate antigen 19–9, *CRP *C-reactive protein

The propensity score distributions showed good overlap between the two groups. After adjustment with IPTW using stabilized weights, the covariates were well balanced between the S-1 + RT and GnP groups, with an absolute SMD of < 0.1 for all covariates (Table 2).

**Table 2:** Patient characteristics after propensity score weighting (stabilized IPTW)

	S-1 + RT	GnP	SMD
Age, years			0.003
< 65	42.7%	42.6%	
0≥ 65	57.3%	57.4%	
Sex			0.021
Male	51.2%	52.2%	
Female	48.9%	47.8%	
ECOG PS			0.031
0	69.7%	71.2%	
1	30.3%	28.8%	
Primary tumor location			0.026
Pancreatic head	58.0%	56.7%	
Pancreatic body or tail	42.0%	43.4%	
Lymph node metastasis			0.003
N0	82.3%	82.2%	
N1	17.7%	17.8%	
Invasion of celiac artery			0.067
No	38.7%	41.9%	
Yes	61.3%	58.1%	
Invasion of superior mesenteric artery			0.024
No	26.8%	27.9%	
Yes	73.2%	72.1%	
CA19-9, U/mL			0.010
< 1,000	75.3%	74.8%	
≥ 1,000	24.7%	25.2%	
Serum albumin, mg/dL			0.013
< 4.0	34.8%	35.5%	
≥ 4.0	65.2%	64.5%	
CRP, mg/dL			0.006
< 0.3	68.2%	68.5%	
≥ 0.3	31.8%	31.5%	

*IPTW* Inverse probability of treatment weighting, *S-1 + RT* S-1 with concurrent radiotherapy, *GnP* Gemcitabine plus nab-paclitaxel, *SMD* Standardized mean difference, *ECOG PS* Eastern Cooperative Oncology Group performance status, *CA19–9* Carbohydrate antigen 19−9, *CRP* C-reactive protein

### Efficacy

**Fig. 2:**
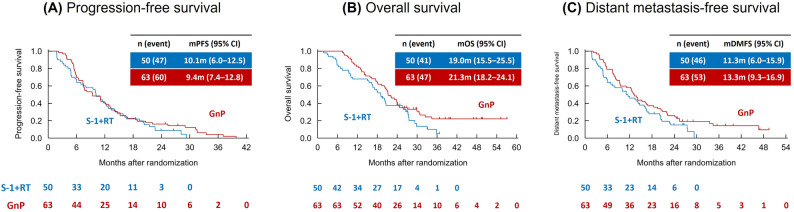
Kaplan–Meier survival curves for progression-free survival (**A**), overall survival (**B**), and distant metastasis-free survival (**C**). S-1 + RT, S-1 with concurrent radiotherapy; GnP, gemcitabine plus nab-paclitaxel; CI, confidence interval

Before conducting analyses using propensity scores, we performed an unadjusted survival comparison. The Kaplan–Meier curves for PFS, OS, and DMFS are shown in Fig. 2. The unadjusted median PFS for S-1 + RT and GnP group was 10.1 vs. 9.4 months (HR for GnP, with S-1 + RT as the reference, 0.83; 95% CI, 0.56–1.24). The unadjusted median OS was 19.0 vs. 21.3 months (HR, 0.68; 95% CI, 0.44–1.03). The unadjusted DMFS was 11.3 vs. 13.3 months (HR, 0.69; 95% CI, 0.46–1.03).

**Fig. 3:**
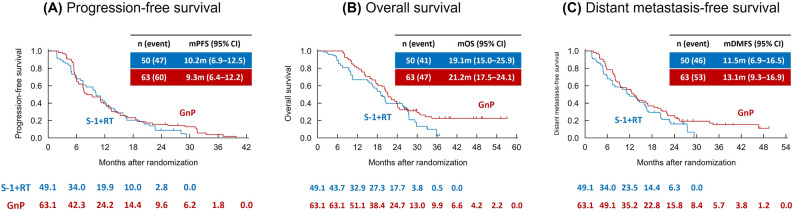
Adjusted Kaplan–Meier survival curves for progression-free survival (**A**), overall survival (**B**), and distant metastasis-free survival (**C**) using IPTW with stabilized weights. IPTW, inverse probability of treatment weighting; S-1 + RT, S-1 with concurrent radiotherapy; GnP, gemcitabine plus nab-paclitaxel; CI, confidence interval

In the IPTW-adjusted analysis using stabilized weights, PFS did not differ significantly between the two groups. Median PFS was 10.2 months for patients treated with S-1 + RT and 9.3 months for those treated with GnP, with an HR of 0.88 (95% CI, 0.60–1.30; two-sided *P* = 0.52) based on the Cox regression model. The median OS and DMFS were 19.1 vs. 21.2 months (HR, 0.73; 95% CI, 0.48–1.11) and 11.5 vs. 13.1 months (HR, 0.73; 95% CI, 0.49–1.08), respectively (Fig. 3; Table 3).

**Table 3:** Comparison of S-1 + RT with GnP on hazard ratios for progression-free survival, overall survival, and distant metastasis-free survival

Models	Sample sizes, No.		Progression-free survival			Overall survival			Distant metastasis-free survival		
	S-1 + RT	GnP	HR (95% CI)		2-sided *P*	HR (95% CI)		2-sided *P*	HR (95% CI)		2-sided *P*
Unadjusted model	50	63	0.83	(0.56–1.24)	0.36	0.68	(0.44–1.03)	0.07	0.69	(0.46–1.03)	0.07
Multivariable adjusted model ^a^	50	63	0.90	(0.59–1.39)	0.64	0.75	(0.47–1.18)	0.21	0.76	(0.49–1.17)	0.21
Propensity score adjusted model ^b^											
Weighting (stabilized IPTW)	50	63	0.88	(0.60–1.30)	0.52	0.73	(0.48–1.11)	0.14	0.73	(0.49–1.08)	0.12
Weighting (IPTW)	50	62	0.87	(0.59–1.29)	0.49	0.72	(0.47–1.10)	0.12	0.72	(0.49–1.07)	0.11
Matching 1:1	41	41	0.91	(0.58–1.40)	0.42	0.68	(0.43–1.09)	0.11	0.77	(0.49–1.21)	0.26
Stratification	50	62	0.83	(0.56–1.24)	0.36	0.68	(0.44–1.03)	0.07	0.69	(0.46–1.03)	0.07
Regression adjustment	50	62	0.91	(0.60–1.37)	0.65	0.75	(0.48–1.17)	0.20	0.73	(0.48–1.10)	0.14

Hazard ratios are presented for GnP, with S-1 + RT as the reference

*S-1 + RT* S-1 with concurrent radiotherapy, *GnP* Gemcitabine plus nab-paclitaxel,* HR* Hazard ratio, *CI* Confidence interval, *IPTW* Inverse probability of treatment weighting

^a^The model was adjusted for baseline age, sex, Eastern Cooperative Oncology Group performance status (ECOG PS), primary tumor location, lymph node metastasis, invasion of celiac artery, invasion of superior mesenteric artery, carbohydrate antigen 19−9 (CA19–9), serum albumin, and C-reactive protein (CRP)

^b^The propensity of receiving gemcitabine plus nab-paclitaxel was estimated using a logistic regression model that included baseline age, sex, ECOG PS, primary tumor location, lymph node metastasis, invasion of celiac artery, invasion of superior mesenteric artery, CA19–9, serum albumin, and CRP

In a sensitivity analysis, after excluding one outlier in the GnP group, the IPTW-adjusted survival analysis revealed that the HRs for PFS, OS, and DMFS of GnP compared with S-1 + RT were 0.87 (95% CI, 0.59–1.29), 0.72 (95% CI, 0.47–1.10), and 0.72 (95% CI, 0.49–1.07), respectively. In the propensity score matching, 41 patients from each group were matched (Supplementary Table 1). The HRs for PFS, OS, and DMFS of GnP compared with S-1 + RT were 0.91 (95% CI, 0.58–1.40), 0.68 (95% CI, 0.43–1.09), and 0.77 (95% CI, 0.49–1.21). In stratified Cox regression analysis, the HRs for PFS, OS, and DMFS of GnP compared with S-1 + RT were 0.83 (95% CI, 0.56–1.24), 0.68 (95% CI, 0.44–1.03), and 0.69 (95% CI, 0.46–1.03). In regression adjustment, the HRs for PFS, OS, and DMFS of GnP compared with S-1 + RT were 0.91 (95% CI, 0.60–1.37), 0.75 (95% CI, 0.48–1.17), and 0.73 (95% CI, 0.48–1.10). In multivariable Cox regression analysis, the estimated HRs for PFS, OS, and DMFS of GnP compared with S-1 + RT were 0.90 (95% CI, 0.59–1.39), 0.75 (95% CI, 0.47–1.18), and 0.76 (95% CI, 0.49–1.17), respectively (Supplementary Table 2). These approaches yielded results similar to those obtained with the IPTW using stabilized weights (Table 3).

Among the treated populations, 40 (80.0%) and 54 patients (85.7%) in the S-1 + RT and GnP groups, respectively, received subsequent treatment. Among them, 77.5% and 38.9% of the S-1 + RT and GnP groups, respectively, received single-agent chemotherapy, including gemcitabine or S-1 monotherapy. Multi-agent chemotherapy was administered to 17.5% and 37.0% of the patients, and chemoradiotherapy was administered to 0% and 13.0% of the patients, respectively (Supplementary Table 3).

### Safety

In all treated populations, the incidence of Grade 3–4 neutrophil count decreased and febrile neutropenia was higher in the GnP than in the S-1 + RT group. S-1 + RT exhibited a higher incidence of gastrointestinal toxicities, such as anorexia, diarrhea, nausea, and vomiting, than GnP (Supplementary Table 4).

## Discussion

In this pooled analysis of two randomized phase II trials, PFS was comparable between the S-1 + RT and GnP groups, whereas OS and DMFS tended to be longer with GnP. These trends were consistent with the results from the IPTW with stabilized weights and its sensitivity analyses, including the multivariable adjusted model and propensity score-adjusted models (IPTW, matching, stratification, and regression adjustment), supporting the robustness of the findings.

The primary endpoint of PFS was comparable between the S-1 + RT and GnP groups; however, OS and DMFS tended to be shorter in the S-1 + RT group. These findings suggest that S-1 + RT may provide effective local control; however, it is less effective than GnP for controlling micrometastatic disease. In the LAP07 trial, chemoradiotherapy was associated with a reduction in local progression compared with chemotherapy (32% vs. 46%), whereas the incidence of distant metastases was higher in the chemoradiotherapy group (60% vs. 44%) [15]. Summarily, these results indicate that the primary benefit of chemoradiotherapy in LAPC may be improved local control, whereas effective systemic chemotherapy remains essential for the suppression of micrometastatic disease.

While radiotherapy may enhance local disease control, the intensity and composition of the chemotherapy delivered as part of chemoradiotherapy may influence treatment outcomes by affecting the control of micrometastatic disease. In the PREOPANC-2 trial, no significant difference in OS was observed between neoadjuvant FOLFIRINOX and neoadjuvant gemcitabine-based chemoradiotherapy in patients with resectable or borderline resectable pancreatic cancer. Differences in outcomes across studies comparing chemoradiotherapy with systemic chemotherapy, including the present analysis and the PREOPANC-2 trial, may therefore be partly attributable to variations in the intensity and composition of chemotherapy incorporated into chemoradiotherapy.

Another reason for the different trends observed in PFS and OS was the influence of subsequent treatments. The patient accrual period for JCOG1106 was from December 2011 to September 2013, whereas that for JCOG1407 was from July 2016 to August 2019. In Japan, FOLFIRINOX and GnP were reimbursed for patients with unresectable pancreatic cancer in December 2013 and December 2014, respectively. The administration of multi-agent chemotherapy as a subsequent treatment was less frequent in the S-1 + RT than in the GnP group (17.5% vs. 37.0%). Considering that GnP and FOLFIRINOX are now available as treatment options after S-1 + RT, the OS in the S-1 + RT group is expected to be longer than that reported in the JCOG1106 trial.

In this exploratory analysis, we compared S-1 + RT and GnP, which are regarded as promising regimens for chemoradiotherapy and systemic chemotherapy, respectively. Nevertheless, treatment strategies for LAPC continue to evolve, and more effective options may become available in the near future. Recently, an increasing number of clinical studies have revealed that radiotherapy enhances the efficacy of immune checkpoint inhibitors (ICIs) [16–18]. Reportedly, combining ICIs with radiotherapy has shown promise in several preclinical models, including pancreatic cancer [19, 20]. A multicenter randomized phase III trial (JCOG1908E) has been ongoing since October 2020 to evaluate the superiority of adding nivolumab to S-1 + RT in patients with LAPC [21]. Advances have been made in systemic chemotherapy. The combination of fluorouracil, leucovorin, liposomal irinotecan, and oxaliplatin (NALIRIFOX) showed superiority over GnP in patients with metastatic pancreatic cancer [22] and is expected to provide meaningful clinical benefits in patients with LAPC. In addition, the CASSANDRA trial demonstrated that a quadruple chemotherapy regimen consisting of cisplatin, nab-paclitaxel, capecitabine, and gemcitabine (PAXG) was superior to modified FOLFIRINOX in patients with resectable and borderline resectable pancreatic cancer [23], suggesting that intensified systemic chemotherapy regimens, including PAXG, may also have a role in the management of LAPC. Furthermore, the PANOVA-3 study revealed significant OS benefits of tumor treating fields plus GnP compared with GnP alone in patients with LAPC [24]. Considering the various advances in the treatment of LAPC, a direct comparison between systemic chemotherapy combined with local treatment and systemic chemotherapy alone remains challenging. Therefore, our study provides valuable evidence to inform treatment decisions regarding the current treatment landscapes.

Our study had some limitations. First, the results were derived from an integrated analysis of two clinical trials rather than from a randomized comparison. Second, the limited sample size made it difficult to generalize the findings to a broader patient population. Third, because JCOG1106 was conducted earlier than JCOG1407, the availability of subsequent treatment options was more limited; therefore, PFS was selected as the primary endpoint in this study. Although this choice minimizes confounding from post-protocol therapies, it may not fully reflect differences in OS, which represents the ultimate clinical benefit. Finally, some patients enrolled in JCOG1407 may have had gastric or duodenal ulcers, which would have made them ineligible for JCOG1106; however, it was not possible to reliably exclude such patients in the present analysis.

## Conclusions

GnP may offer advantages in suppressing micrometastatic disease, whereas S-1 + RT may provide benefits in local disease control. These findings suggest that both approaches represent important and complementary therapeutic options for LAPC. Prospective randomized studies are warranted to determine the optimal initial strategy.

## Supplementary Information

Supplementary Material 1.

## Data Availability

The datasets used and/or analyzed during the current study are available from the corresponding author on reasonable request.

## Abbreviations

CA19–9: Carbohydrate antigen 19–9
CI: Confidence interval
CRP: C-reactive protein
DMFS: Distant metastasis-free survival
ECOG PS: Eastern Cooperative Oncology Group performance status
GnP: Gemcitabine plus nab-paclitaxel
ICIs: Immune checkpoint inhibitors
IPTW: Inverse probability of treatment weighting
JCOG: Japan Clinical Oncology Group
LAPC: Locally advanced pancreatic cancer
OS: Overall survival
PFS: Progression-free survival
SMD: Standardized mean difference
S-1 + RT: S-1 with concurrent radiotherapy
