# Supplementary material for: Comparison of chemoradiotherapy and gemcitabine plus nab-paclitaxel for locally advanced pancreatic cancer: an integrated analysis of two randomized phase II trials (JCOG2408A)
Source: BMC Cancer. 2026 Feb 10;26:363. doi: 10.1186/s12885-026-15699-8 (PMC12998374; doi:10.1186/s12885-026-15699-8)
Supplement: Supplementary file 1 — Supplementary Material 1. [file 12885_2026_15699_MOESM1_ESM.docx]

# **Supplementary Table 1** Patient characteristics in propensity score matching

|  | S-1 + RT (n=41) | | GnP (n=41) | | SMD |
| --- | --- | --- | --- | --- | --- |
| Age, years |  |  |  |  | 0.049 |
| < 65 | 19 | (46.3) | 18 | (43.9) |  |
| ≥ 65 | 22 | (53.7) | 23 | (56.1) |  |
| Sex |  |  |  |  | 0.049 |
| Male | 20 | (48.8) | 21 | (51.2) |  |
| Female | 21 | (51.2) | 20 | (48.8) |  |
| ECOG PS |  |  |  |  | 0.161 |
| 0 | 31 | (75.6) | 28 | (68.3) |  |
| 1 | 10 | (24.4) | 13 | (31.7) |  |
| Primary tumor location |  |  |  |  | 0.246 |
| Pancreatic head | 26 | (63.4) | 21 | (51.2) |  |
| Pancreatic body or tail | 15 | (36.6) | 20 | (48.8) |  |
| Lymph node metastasis |  |  |  |  | 0.062 |
| N0 | 33 | (80.5) | 32 | (78.0) |  |
| N1 | 8 | (19.5) | 9 | (22.0) |  |
| Invasion of celiac artery |  |  |  |  | 0.198 |
| No | 18 | (43.9) | 14 | (34.1) |  |
| Yes | 23 | (56.1) | 27 | (65.9) |  |
| Invasion of superior mesenteric artery | |  |  |  | 0.056 |
| No | 9 | (22.0) | 10 | (24.4) |  |
| Yes | 32 | (78.0) | 31 | (75.6) |  |
| CA19–9, U/mL |  |  |  |  | 0.056 |
| < 1,000 | 31 | (75.6) | 30 | (75.6) |  |
| ≥ 1,000 | 10 | (24.4) | 11 | (24.4) |  |
| Serum albumin, mg/dL |  |  |  |  | 0.051 |
| < 4.0 | 14 | (34.1) | 13 | (31.7) |  |
| ≥ 4.0 | 27 | (65.9) | 28 | (68.3) |  |
| CRP, mg/dL |  |  |  |  | 0.000 |
| < 0.3 | 28 | (68.3) | 28 | (68.3) |  |
| ≥ 0.3 | 13 | (31.7) | 13 | (31.7) |  |

Data are presented as n (%)

S-1 + RT, S-1 with concurrent radiotherapy; GnP, gemcitabine plus nab-paclitaxel; SMD, standardized mean difference; ECOG PS, Eastern Cooperative Oncology Group performance status; CA19–9, carbohydrate antigen 19–9; CRP, C-reactive protein.

# **Supplementary Table 2.** Multivariable analysis of progression-free survival, overall survival, and distant metastasis-free survival

|  |  | Progression-free survival | |  | Overall survival | |  | Distant metastasis-free survival | |
| --- | --- | --- | --- | --- | --- | --- | --- | --- | --- |
| Factors |  | HR (95% CI) | 2-sided P |  | HR (95% CI) | 2-sided P |  | HR (95% CI) | 2-sided P |
| Treatment | GnP (vs. S-1 + RT) | 0.90 (0.59–1.39) | 0.64 |  | 0.75 (0.47–1.18) | 0.21 |  | 0.76 (0.49–1.17) | 0.21 |
| Age, years | ≥ 65 (vs. < 65) | 1.01 (0.65–1.59) | 0.96 |  | 1.11 (0.69–1.78) | 0.67 |  | 1.17 (0.74–1.84) | 0.50 |
| Sex | Female (vs. Male) | 1.27 (0.82–1.96) | 0.29 |  | 1.59 (0.98–2.59) | 0.06 |  | 1.21 (0.77–1.88) | 0.41 |
| ECOG PS | 1 (vs. 0) | 1.80 (1.12–2.88) | 0.01 |  | 1.52 (0.93–2.48) | 0.09 |  | 1.55 (0.97–2.49) | 0.07 |
| Primary tumor location | Pb, Pt (vs. Ph) | 1.07 (0.64–1.78) | 0.81 |  | 1.16 (0.66–2.02) | 0.61 |  | 1.13 (0.66–1.93) | 0.65 |
| Lymph node metastasis | N1 (vs. N0) | 1.17 (0.67–2.06) | 0.59 |  | 1.09 (0.60–1.99) | 0.77 |  | 1.47 (0.84–2.55) | 0.18 |
| Invasion of CA | Yes (vs. No) | 1.09 (0.67–1.78) | 0.73 |  | 0.90 (0.53–1.55) | 0.71 |  | 0.92 (0.56–1.51) | 0.74 |
| Invasion of SMA | Yes (vs. No) | 1.27 (0.75–2.14) | 0.37 |  | 1.18 (0.69–2.03) | 0.55 |  | 1.47 (0.87–2.49) | 0.15 |
| CA19–9, IU/mL | ≥ 1,000 (vs. < 1,000) | 1.10 (0.67–1.79) | 0.72 |  | 1.20 (0.71–2.02) | 0.50 |  | 1.19 (0.74–1.94) | 0.47 |
| Serum albumin, mg/dL | ≥ 4.0 (vs. < 4.0) | 1.23 (0.76–1.99) | 0.41 |  | 0.93 (0.56–1.55) | 0.79 |  | 1.23 (0.75–2.03) | 0.42 |
| CRP, mg/dL | ≥ 0.3 (vs. < 0.3) | 1.49 (0.95–2.35) | 0.08 |  | 1.21 (0.74–1.98) | 0.44 |  | 1.24 (0.77–2.01) | 0.38 |

HR, hazard ratio; CI, confidence interval; GnP, gemcitabine plus nab-paclitaxel; S-1 + RT, S-1 with concurrent radiotherapy; ECOG PS, Eastern Cooperative Oncology Group performance status; CA, celiac artery; SMA, superior mesenteric artery; CA19–9, carbohydrate antigen 19–9; CRP, C-reactive protein.

# **Supplementary Table 3.** Subsequent anti-cancer treatment (all treated populations)

|  | S-1 + RT (n=50) | | | GnP (n=63) | | |
| --- | --- | --- | --- | --- | --- | --- |
| Subsequent treatment |  |  | |  |  | |
| Yes | 40 | (80.0) | | 54 | (85.7) | |
| No | 10 | (20.0) | | 9 | (14.3) | |
|  |  |  | |  |  | |
| Details among treated |  |  | |  |  | |
| Single-agent chemotherapy | 31 | (77.5) | | 21 | (38.9) | |
| Gemcitabine | 17 | | (42.5) | 12 | | (22.2) |
| S-1 | 14 | | (35.0) | 9 | | (16.7) |
| Multi-agent chemotherapy | 7 | (17.5) | | 20 | (37.0) | |
| Fluoropyrimidine-based | 5 | | (12.5) | 16 | | (29.6) |
| Gemcitabine-based | 2 | | (5.0) | 4 | | (7.4) |
| Chemoradiotherapy | 0 |  | | 7 | (13.0) | |
| Conversion surgery | 2 | (5.0) | | 6 | (11.1) | |

Data are presented as n (%).

S-1 + RT, S-1 with concurrent radiotherapy; GnP, gemcitabine plus nab-paclitaxel.

# **Supplementary Table 4.** A summary of adverse events (all treated populations)

|  | S-1 + RT (n=50) | | | |  | GnP (n=63) | | | |
| --- | --- | --- | --- | --- | --- | --- | --- | --- | --- |
|  | All grade | | Grade 3–4 | |  | All grade | | Grade 3–4 | |
| White blood cell decreased | 47 | (94.0) | 31 | (62.0) |  | 52 | (82.5) | 28 | (44.4) |
| Neutrophil count decreased | 46 | (92.0) | 27 | (54.0) |  | 61 | (96.8) | 50 | (79.4) |
| Anemia | 50 | (100) | 9 | (18.0) |  | 54 | (85.7) | 12 | (19.0) |
| Platelet count decreased | 50 | (100) | 5 | (10.0) |  | 59 | (93.7) | 2 | (3.2) |
| Blood bilirubin increased | 17 | (34.0) | 4 | (8.0) |  | 9 | (14.3) | 2 | (3.2) |
| AST increased | 44 | (88.0) | 7 | (14.0) |  | 47 | (74.6) | 8 | (12.7) |
| ALT increased | 47 | (94.0) | 7 | (14.0) |  | 51 | (81.0) | 10 | (15.9) |
| Febrile neutropenia | 0 |  | 0 |  |  | 3 | (4.8) | 3 | (4.8) |
| Fatigue | 33 | (66.0) | 4 | (8.0) |  | 39 | (61.9) | 0 |  |
| Anorexia | 44 | (88.0) | 8 | (16.0) |  | 37 | (58.7) | 4 | (6.3) |
| Diarrhea | 23 | (46.0) | 3 | (6.0) |  | 25 | (39.7) | 1 | (1.6) |
| Mucositis oral | 6 | (12.0) | 0 |  |  | 13 | (20.6) | 0 |  |
| Nausea | 40 | (80.0) | 4 | (8.0) |  | 19 | (30.2) | 4 | (6.3) |
| Vomiting | 25 | (50.0) | 1 | (2.0) |  | 8 | (12.7) | 2 | (3.2) |
| Biliary infection | 10 | (20.0) | 10 | (20.0) |  | 15 | (23.8) | 15 | (23.8) |
| Pneumonitis | 3 | (6.0) | 2 | (4.0) |  | 6 | (9.5) | 1 | (1.6) |
| Dysgeusia | 17 | (34.0) | – |  |  | 15 | (23.8) | – |  |
| Gastric/duodenal hemorrhage | 5 | (10.0) | 5 | (10.0) |  |  |  |  |  |
| Gastric/duodenal ulcer | 3 | (6.0) | 3 | (6.0) |  |  |  |  |  |
| Peripheral sensory neuropathy |  |  |  |  |  | 46 | (73.0) | 23 | (36.5) |
| Alopecia |  |  |  |  |  | 41 | (65.1) | – |  |

Data are presented as n (%).

S-1 + RT, S-1 with concurrent radiotherapy; GnP, gemcitabine plus nab-paclitaxel; AST, aspartate aminotransferase; ALT, alanine aminotransferase
